# Microbiome and metabolomic changes associated with HPV clearance in women undergoing local excisional treatment for cervical intraepithelial neoplasia

**DOI:** 10.1128/msystems.00511-25

**Published:** 2025-05-20

**Authors:** Xiaowen Pu, Jingjing Wang, Zhengrong Gu, Hongfeng Ao, Chao Li

**Affiliations:** 1 Department of Gynecology, Shanghai First Maternity and Infant Hospital, School of Medicine, Tongji University 481875, Shanghai, China; 2 Department of Pathology, Shanghai Fengxian District Central Hospital, Shanghai Jiao Tong University Affiliated Sixth People’s Hospital South Campus 385686 , Shanghai, China; 3 Shanghai Key Laboratory of Maternal Fetal Medicine, Shanghai Institute of Maternal-Fetal Medicine and Gynecologic Oncology, Clinical and Translational Research Center, Shanghai First Maternity and Infant Hospital, School of Medicine, Tongji University 481875, Shanghai, China; University of Massachusetts Amherst, Amherst, Massachusetts, USA

**Keywords:** cervical intraepithelial neoplasia, microbiome, metabolome, HPV

## Abstract

Cervical intraepithelial neoplasia (CIN) is a common gynecological condition often associated with persistent human papillomavirus (HPV) infections. Although the loop electrosurgical excision procedure (LEEP) is effective in removing lesions, some patients remain HPV positive post-treatment. In this prospective study, we enrolled reproductive-age women diagnosed with HPV-related CIN and employed a multi-omics analysis of cervicovaginal secretion and cervical tissue microbiomes, alongside non-targeted and targeted metabolomic assessments. We aim to explore the role of the cervicovaginal and intratissue microbiota and associated metabolites on HPV clearance following LEEP. We observed significant shifts in bacterial diversity and composition in both cervicovaginal secretion and cervical tissue samples. Notably, distinct bacterial species, such as *Lactobacillus* and certain anaerobes (e.g., *Prevotella bivia*), were correlated with HPV clearance post-LEEP. Metabolomic profiling revealed that the HPV-cleared group exhibited elevated acetic acid levels and significant alterations in glycerophospholipid metabolism, suggesting a potential role in promoting HPV clearance. Correlation analyses demonstrated significant associations between altered bacteria and metabolites with HPV status, with models incorporating both achieving high predictive accuracy. Overall, our findings highlight the importance of the cervicovaginal and intratissue microbiomes and metabolites in facilitating HPV clearance, suggesting potential therapeutic targets for patients with CIN.

**IMPORTANCE:**

The clearance of human papillomavirus (HPV) after local excisional treatment for cervical intraepithelial neoplasia is crucial for patient health. This study reveals significant alterations in the cervicovaginal secretion and cervical tissue microbiomes, alongside metabolomic changes, which are associated with HPV clearance. Through a comprehensive multi-omics approach, we identified specific bacterial species and metabolic changes that correlate with successful HPV clearance post-loop electrosurgical excision procedure. Notably, the presence of beneficial *Lactobacillus* species and elevated levels of acetic acid linked to glycerophospholipid metabolism emerged as potential biomarkers for HPV status, suggesting that these factors play a pivotal role in improving treatment outcomes. These findings highlight the potential for microbiome-targeted therapies to enhance HPV clearance and provide insights into the microbial and metabolic mechanisms involved in cervical health.

## INTRODUCTION

Cervical intraepithelial neoplasia (CIN) is a prevalent gynecological condition often linked to persistent human papillomavirus (HPV) infections, characterized by abnormal cell growth on the cervix (1). The primary treatment for CIN involves the loop electrosurgical excision procedure (LEEP), which effectively removes lesions in most cases (2). However, some patients do not achieve HPV negativity even after lesion removal (2, 3). This persistent infection may increase the risk of CIN recurrence or progression to cervical cancer (1) and could further adversely affect pregnancy outcomes (4). Research has indicated that LEEP can alter the cervicovaginal microenvironment, impacting patient outcomes (2, 3, 5–9). While there have been efforts to characterize the cervicovaginal bacterial communities associated with HPV (10–12), the causal relationships related to HPV clearance failures remain underexplored.

The intratissue microbiota has gained recognition as a crucial component of the tissue microenvironment, particularly concerning cancers arising from mucosal areas like the cervical mucosa (13, 14). Recent investigations into the tissue microbiome aim to elucidate the interactions between microorganisms and their role within tissue environments (15, 16). Most studies on the cervical microbiome have concentrated on the influence of microbial populations in the vaginas and cervix on cervical cancer pathogenesis (17–19), with less focus on the intratissue microbiome’s role in CIN development and HPV infection (20, 21). Understanding the interplay between the intratissue microbiome and HPV clearance post-LEEP is vital for prognostic assessments of CIN. Moreover, the precise relationship between cervicovaginal and intratissue microbiomes remains poorly understood.

In addition to microbiota, the cervicovaginal microenvironment includes host-derived and microbially produced metabolites that may influence HPV status. HPV infections can disrupt the balance of the cervicovaginal microbiome, leading to metabolic changes (16). Studies have highlighted differences in the metabolomic profiles of HPV-positive versus HPV-negative women, revealing specific alterations in substances such as biogenic amines and fatty acids (22, 23). Other studies have revealed the metabolomic changes present in HPV-positive individuals, particularly within dysbiotic microbial communities (22, 24–27). Nevertheless, the role of these metabolites in HPV clearance post-LEEP remains largely unexamined. Additionally, the intricate interactions among cervicovaginal and intratissue microbiota, cervicovaginal metabolites, and HPV clearance merit further research.

This prospective study enrolled reproductive-age women diagnosed with CIN, employing a multi-omics approach that included analyses of cervicovaginal secretion and cervical tissue microbiomes, alongside non-targeted and targeted metabolomic assessments focusing on short-chain fatty acids (SCFAs). The objective was to compare the microbiota of HPV-cleared versus non-cleared CIN patients post-LEEP and identify specific microbiota and metabolites associated with HPV clearance. The findings aim to provide insights into the mechanisms of CIN progression and potential therapeutic strategies for enhancing HPV clearance.

## RESULTS

### Participant population

**Fig 1:**
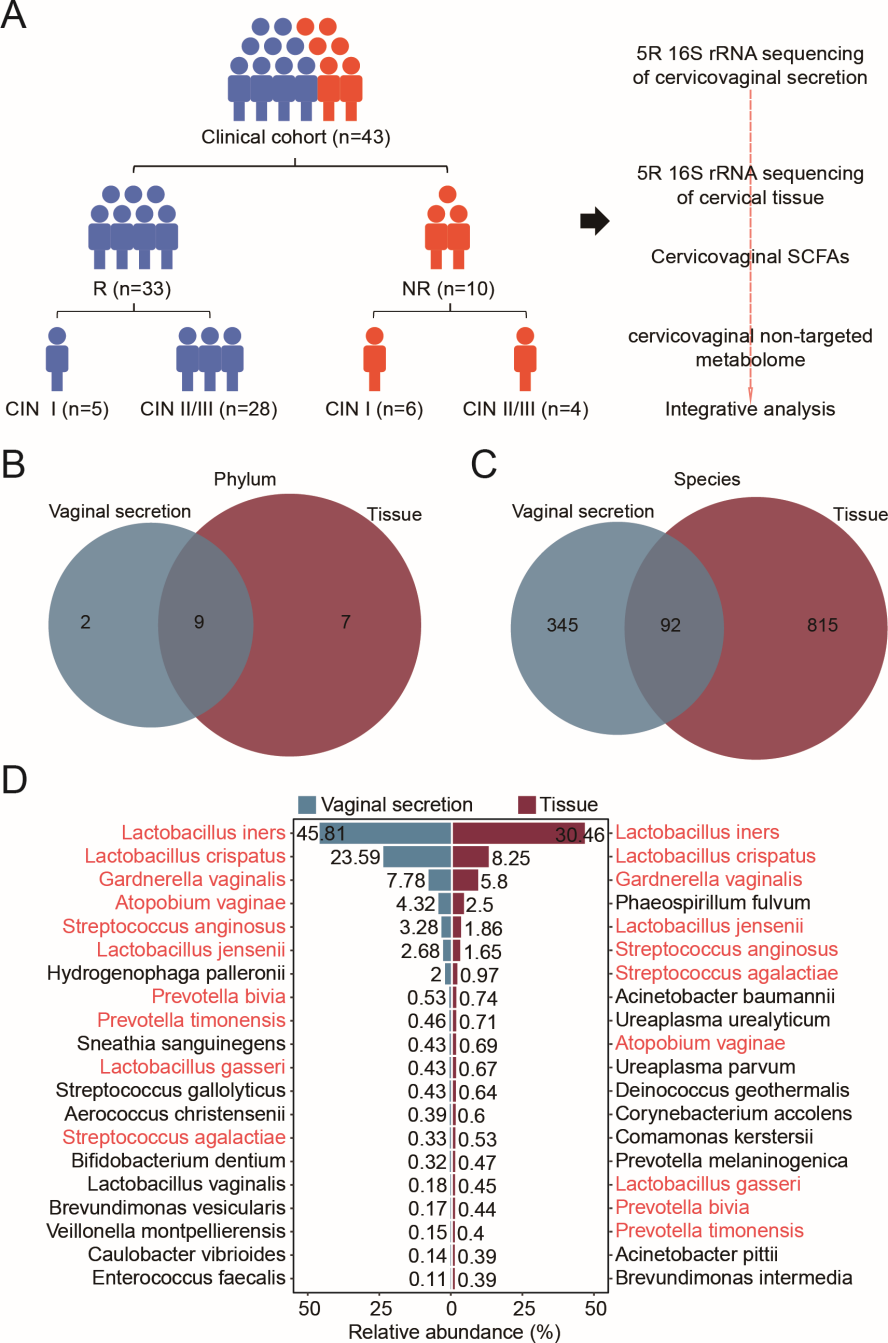
Comparison of the results of the 5R 16S rRNA gene sequencing from cervicovaginal secretions and cervical tissue samples. (**A**) Study workflow illustrating that participants underwent sequencing of cervicovaginal secretion and cervical tissue 5R 16S rRNA, along with targeted metabolomic analysis of SCFAs and non-targeted metabolomics, followed by bioinformatic integration of microbiome and metabolome data. (B and C) Venn diagrams indicating the distribution of shared and unique microbiota at the phylum level (**B**) and species level (**C**). (**D**) A butterfly diagram showcasing the top 20 bacterial species from cervicovaginal secretion and cervical tissue samples, with shared species highlighted in red.

Among the 43 participants diagnosed with HPV-related CIN (11 with CIN I and 32 with CIN II/III), all underwent LEEP. The patients were classified into two groups: R (HPV cleared) and NR (HPV non-cleared) (Fig. 1A). The general characteristics of the participants are summarized in Table 1. Significant alterations in cervicovaginal pH were observed in both the NR and R groups after the LEEP procedure. Regarding the overall demographic characteristics, the only significant difference observed was the number of childbirths (*P* = 0.005).

**TABLE 1:** Baseline clinical characteristics of the women included^*a*^

Parameter	SIL-NR (***n*** = 10)	SIL-R (*n* = 33)	*P* value		
			NR-post vs NR-pre	R-post vs R-pre	SIL-NR vs SIL-R
Age (years)	36 ± 7.78	34 ± 7.39			0.4304
BMI (kg/m^2^)	22.81 ± 2.68	22.60 ± 2.88			0.3605
Cervicovaginal pH			0.0018	0.0000	
Pre-T	7.18 ± 1.35	6.97 ± 1.14			0.6332
Post-T	5.3 ± 0.90	5.14 ± 0.61			0.5090
HPV, *N* (%)					0.2477
High risk	10 (100)	29 (87.9)			
Medium-low risk	0 (0.0)	4 (12.1)			
Marital status, *N* (%)					0.0679
Never married	0 (0.0)	8 (24.2)			
Married	10 (100)	22 (66.7)			
Vaginal discharge, *N* (%)					0.8537
Normal	9 (90.0)	29 (87.9)			
Unusual	1 (10.0)	4 (12.1)			
Sexual partners, *N* (%)					0.1838
1–5	8 (80.0)	31 (93.9)			
>5	2 (20.0)	2 (6.1)			
Age at sexual debut, *N* (%)					0.3904
<20	1 (10.0)	10 (30.3)			
20–30	8 (80.0)	19 (57.6)			
>30	1 (10.0)	4 (12.1)			
No. of childbirths, *N* (%)					0.0049
0	3 (30.0)	8 (24.2)			
1	4 (40.0)	24 (72.7)			
2	2 (20.0)	1 (3.0)			
3	1 (10.0)	0 (0.0)			
No. of abortions, *N* (%)					0.5647
0	4 (40.0)	15 (45.4)			
1	3 (30.0)	13 (39.4)			
2	3 (30.0)	5 (15.2)			
Contraception, *N* (%)					0.6402
Nil	3 (30.0)	6 (18.2)			
Condoms	7 (70.0)	26 (78.8)			
IUCD/IUD/Mirena/contraceptive	0 (0.0)	1 (3.0)			

^*a*^ The data are expressed as the number or mean ± SD. BMI, body mass index. *P*-values for categorical and continuous variables were obtained from the chi-square test and *t*-test, respectively.

For the multiomics analysis, we collected 326 samples from the 43 patients. This analysis encompassed: (i) bacterial 5R 16S rRNA gene sequencing of 43 paired cervicovaginal secretion samples; (ii) bacterial 5R 16S rRNA gene sequencing of 34 paired cervical tissue samples; (iii) targeted metabolomics focusing on SCFAs from 43 paired cervicovaginal secretion samples; and (iv) non-targeted metabolomics conducted on 43 paired cervicovaginal secretion samples (Fig. 1A).

### Overview of the results of the 5R 16S rRNA obtained from cervicovaginal secretion and cervical tissue samples

At the phylum level, we identified a total of 11 phyla in cervicovaginal secretions and 16 in cervical tissue samples, with 9 phyla common to both (Fig. 1B). At the species level, 437 bacterial species were found in cervicovaginal secretions, compared to 907 in cervical tissue (Fig. 1C). Among these, only 92 species (7.3%) were shared; 345 species (27.6%) were exclusive to cervicovaginal secretions, while 815 species (65.1%) were unique to cervical tissue. An analysis of the top 20 most abundant bacterial species showed that half were present in both sample types (Fig. 1D). Notably, species such as *Lactobacillus iners*, *Lactobacillus crispatus*, *Lactobacillus jensenii*, and *Lactobacillus gasseri*, along with harmful anaerobic bacteria, including *Gardnerella vaginalis*, *Atopobium vaginae*, *Streptococcus anginosus*, *Streptococcus agalactiae*, and *Prevotella timonensis*, were the most prominent. This aligns with findings from previous studies (16, 28).

### Bacterial diversity and composition in cervicovaginal secretions before and after LEEP surgery

**Fig 2:**
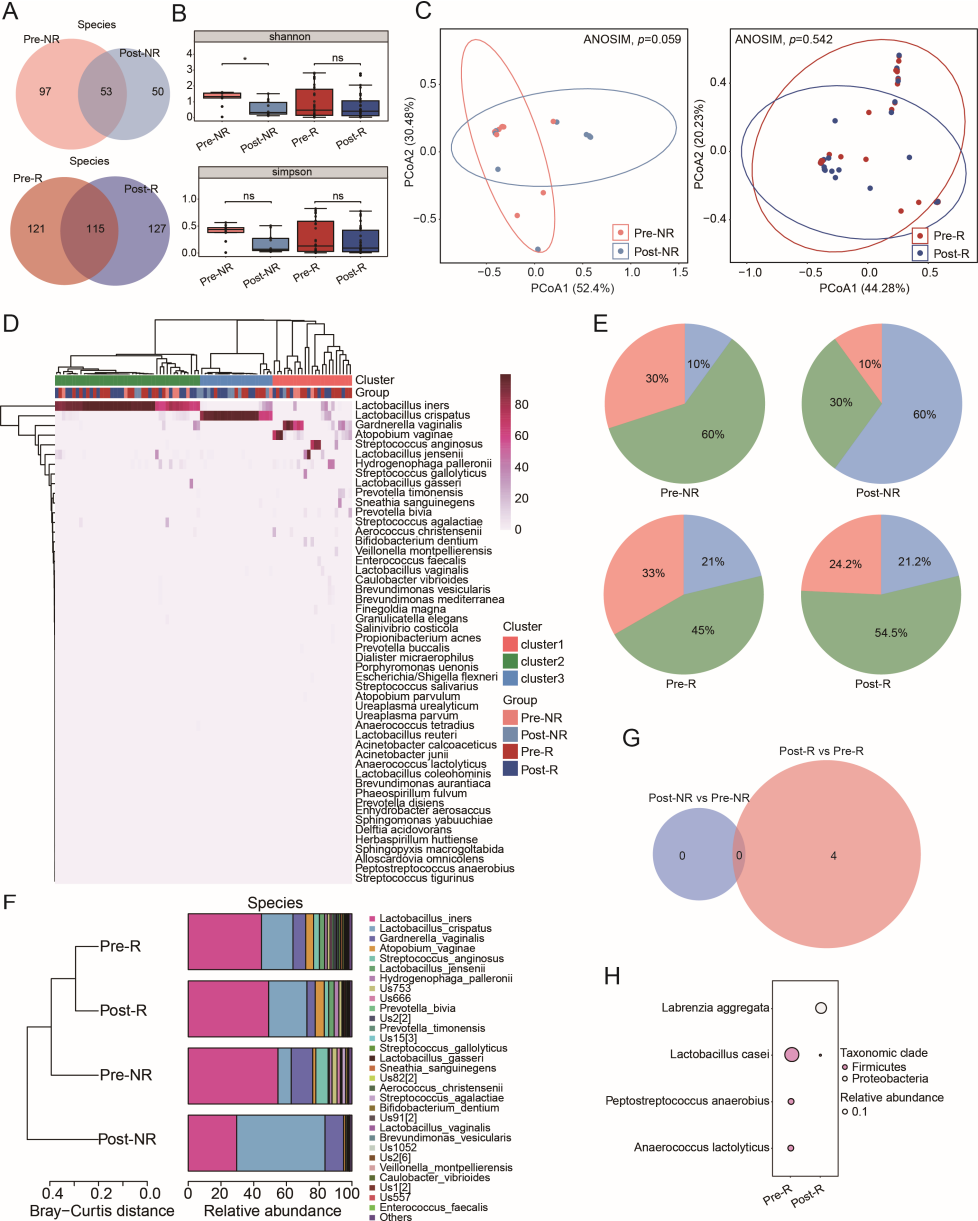
Analysis of bacterial diversity and composition of cervicovaginal secretions pre- and post-LEEP surgery. (**A**) Venn diagrams comparing bacterial species before and after LEEP, specifically between pre-NR and post-NR (top) and pre-R and post-R (bottom). Pre, samples collected before LEEP surgery; post, samples collected after LEEP surgery. (**B**) Alpha diversity measures, using Shannon (top) and Simpson (bottom) indices, comparing pre- and post-LEEP samples for both groups. *P* values derived from the Mann-Whitney *U* test. (**C**) Beta diversity analyzed through PCoA based on Bray-Curtis distances between pre-NR and post-NR (left), and between pre-R and post-R (right), with significance assessed using ANOSIM. (**D**) Heatmap depicting the top 50 bacterial species across 86 samples. (**E**) Visualization of bacterial clusters within the analyzed groups. (**F**) Hierarchical clustering on the average relative proportions of the 30 most abundant species among the four groups based on Bray-Curtis distances. (**G**) A Venn diagram presenting unique differentially abundant bacterial species (DABs) specific to the R group (Mann-Whitney *U* test, *P* < 0.05). (**H**) A bubble diagram showcasing the four DABs exclusive to the R group.

The amplicon-based 5R 16S rRNA gene sequencing (15) was employed to evaluate the effect of local excisional treatment on the taxonomic composition of cervicovaginal secretions. At the phylum level, the majority remained consistent following the surgical procedure (Fig. S1A). At the species level, we identified 200 bacterial species in the NR group and 363 in the R group (Fig. 2A). Notably, 50 species (25.0%) were unique to the NR group post-surgery, while the R group exhibited 127 unique species (35.0%) after the procedure. Alpha diversity analysis revealed no significant differences in the Shannon, Simpson, Observed features, or Chao1 indices for both groups before and after surgery, except for a significant change in the Shannon index between pre-NR and post-NR (Fig. 2B; Fig. S1B). Furthermore, principal coordinates analysis (PCoA) based on Bray-Curtis distance showed no significant differences between the post-NR and pre-NR groups or the post-R and pre-R groups (ANOSIM, *P* = 0.059 and *P* = 0.542, respectively) (Fig. 2C).

To explore variations in bacterial community types, unsupervised clustering categorized bacteria into three distinct clusters based on their composition and relative abundances (Fig. 2D; Table S1). Cluster I was characterized by a reduced number of *Lactobacillus* species and a greater diversity of anaerobic or facultative anaerobic bacteria, while clusters II and III were predominantly composed of *L. iners* and *L. crispatus*, respectively. After LEEP surgery, the proportion of *Lactobacillus*-dominant clusters II and III increased in both NR and R groups (Fig. 2E). Bacterial classification analysis revealed that the distribution of dominant bacteria varied according to surgical status at the phylum level and HPV status at the species level, while also showing a decrease in diversity within the post-surgery group (Fig. 2F; Fig. S1C). Additional statistical analysis using the Mann-Whitney *U* test identified no differentially abundant bacterial species (DABs; *P* < 0.05) in the NR group, while four DABs were found in the R group (Fig. 2G). These four bacterial species—*Labrenzia aggregata*, *Lacticaseibacillus casei*, *Peptostreptococcus anaerobius*, and *Anaerococcus lactolyticus*—may be associated with the clearance of HPV (Fig. 2H).

### Bacterial diversity and composition in cervical tissues before and after LEEP surgery

**Fig 3:**
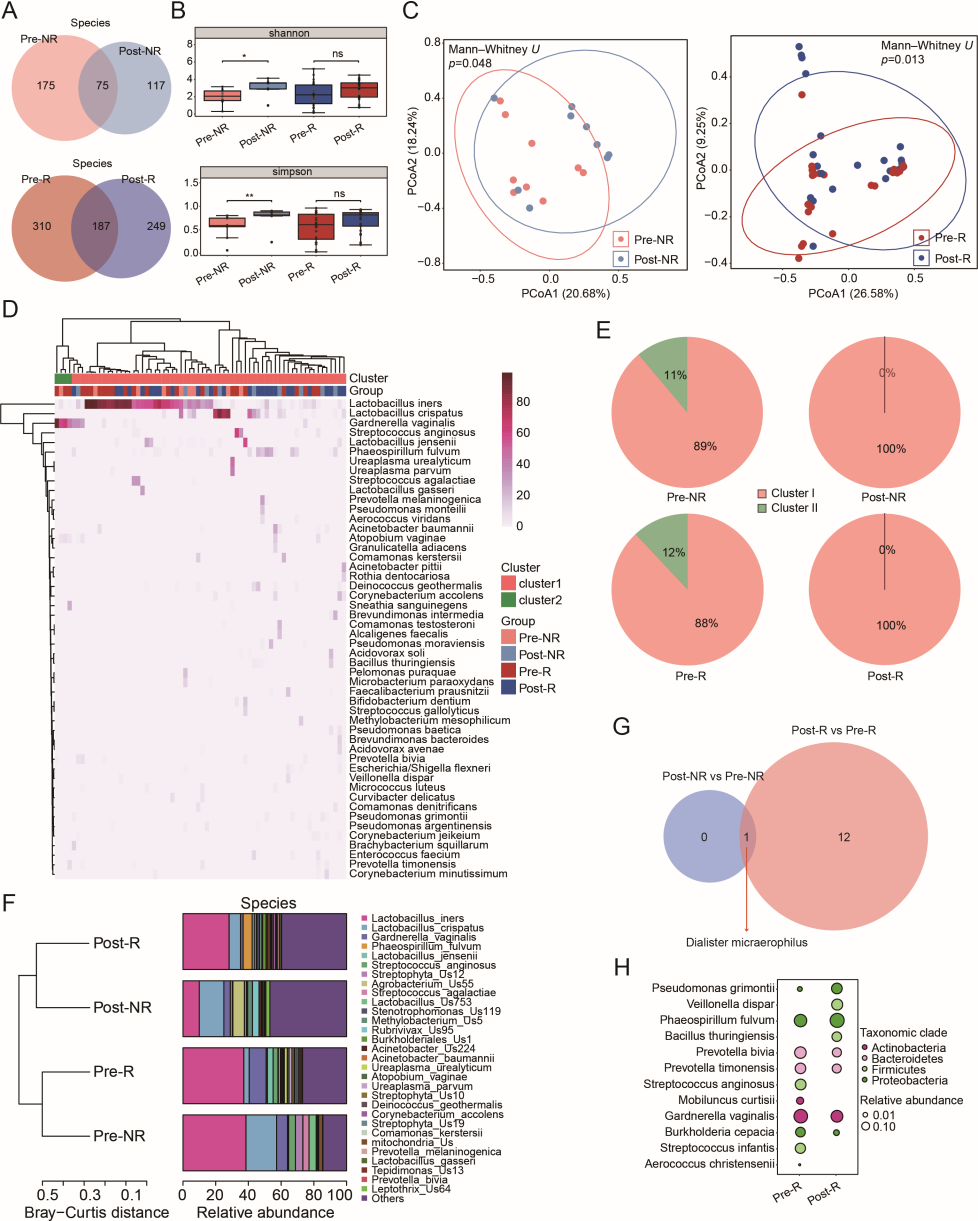
Analysis of bacterial diversity and composition of cervical tissue pre- and post-LEEP surgery. (**A**) Venn diagrams comparing bacterial species before and following LEEP, specifically for pre-NR vs post-NR (top) and pre-R vs post-R (bottom). (**B**) Assessment of alpha diversity via Shannon (top) and Simpson (bottom) indices between the sample pairs, using the Mann-Whitney *U* test for statistical analysis. (**C**) Beta diversity determined through PCoA based on Bray-Curtis distances between pre-NR and post-NR (left), as well as between pre-R and post-R (right), with significance evaluated by ANOSIM. (**D**) Heatmap illustrating the top 50 bacterial species across 68 samples. (**E**) Visualization of bacterial clusters within the assessed groups. (**F**) Hierarchical clustering on the average proportions of the 30 most abundant species among the four groups based on Bray-Curtis distances. (**G**) A Venn diagram representing unique DABs for the R group (Mann-Whitney *U* test, *P* < 0.05). (**H**) A bubble diagram illustrating 11 DABs identified within the R group.

To assess the changes in taxonomic composition within cervical tissues, we conducted 5R 16S rRNA gene sequencing. Similar to the findings in cervicovaginal secretions, the predominant phyla remained consistent post-surgery (Fig. S1D). At the species level, we identified a total of 367 bacterial species in the NR group and 746 in the R group (Fig. 3A). Notably, 117 species (31.9%) were unique to the NR group following surgery, while the R group exhibited 249 unique species (33.4%). Alpha diversity analysis revealed significant differences in the Shannon and Simpson indices for the NR group pre- and post-surgery; in contrast, no significant differences were observed in the R group (Fig. 3B; Fig. S1E). Principal coordinates analysis, based on Bray-Curtis distances, displayed marked differences between the post-NR and pre-NR groups as well as the post-R and pre-R groups (ANOSIM, *P* = 0.048 and *P* = 0.013, respectively) (Fig. 3C).

To explore variations in bacterial community types, we performed unsupervised clustering, categorizing bacteria into two distinct clusters (Fig. 3D; Table S2). Cluster I was primarily composed of *Lactobacillus* species, including *L. iners*, *L. crispatus*, and *L. jensenii*, while Cluster II was mainly characterized by *G. vaginalis*. Following LEEP surgery, the bacterial composition in nearly all patients shifted toward the *Lactobacillus*-dominant Cluster I (Fig. 3E). Analysis of bacterial classification indicated that the distribution of the dominant bacteria was associated with surgical status at both the phylum and species levels (Fig. 3F; Fig. S1F). Further statistical evaluation using the Mann-Whitney *U* test revealed 1 and 13 DABs in the NR and R groups, respectively, after surgery (Fig. 3G). Notably, the 12 bacterial species unique to the R group might be correlated with HPV clearance (Fig. 3H), and interestingly, these species were entirely distinct from those identified in cervicovaginal secretions (Fig. 2H).

### LEEP surgery changes the cervicovaginal metabolomic profiles

**Fig 4:**
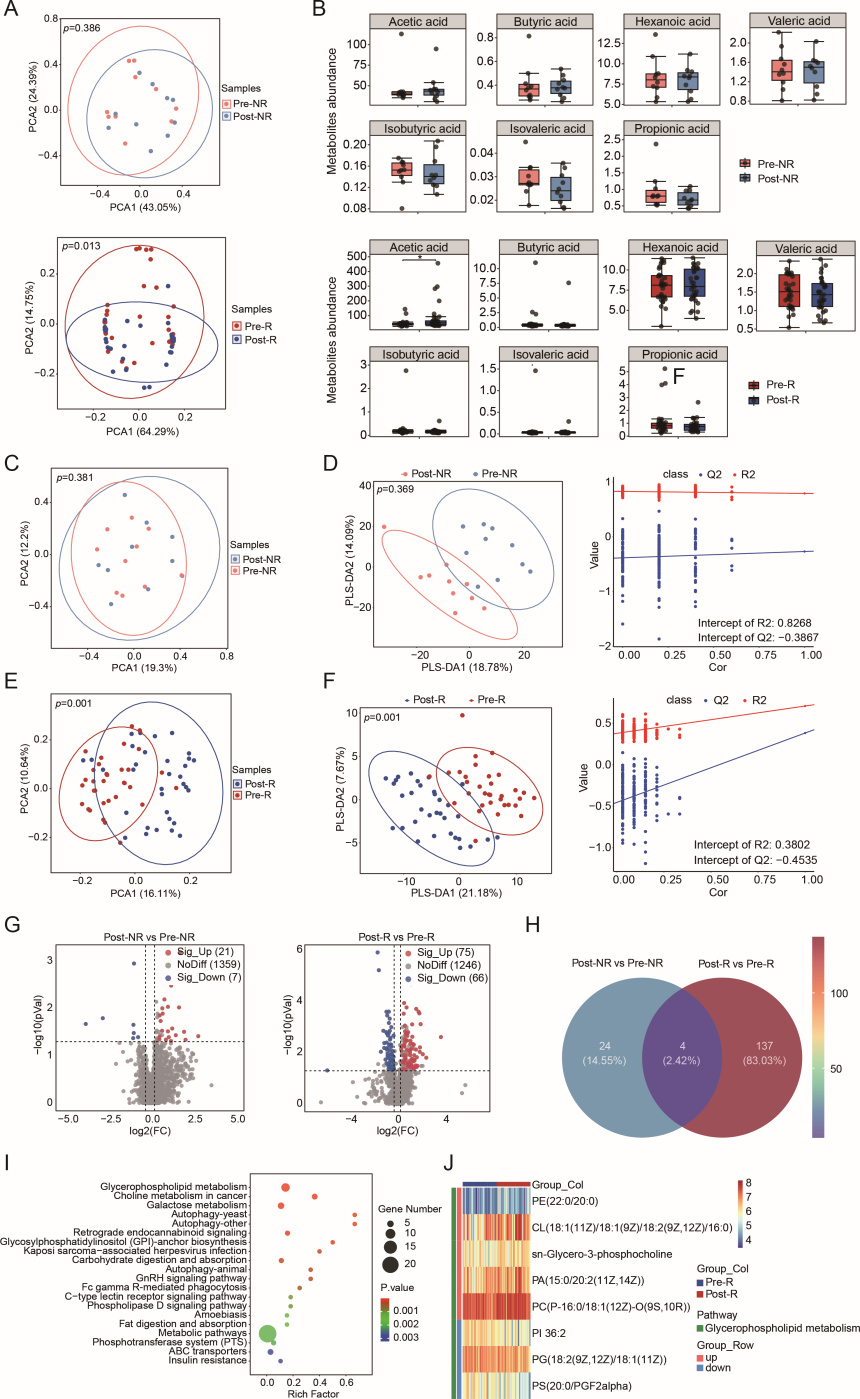
Metabolic profiling of the cervicovaginal metabolome pre- and post-LEEP surgery. (**A**) PCA plots based on Bray-Curtis distances for SCFAs comparing pre-NR vs post-NR (top) and pre-R vs post-R (bottom), with significance derived from ANOSIM. (**B**) Box plots depicting SCFA levels for comparing pre-NR and post-NR (top), as well as pre-R and post-R (bottom), before and after LEEP. (C and E) PCA plots for non-targeted metabolomics relating to pre-NR vs post-NR (**C**) and pre-R vs post-R (**E**), assessed for significance via ANOSIM. (D and F) Partial least squares discriminant analysis was utilized for comparisons between pre-NR and post-NR (**D**), alongside the pre-R and post-R (**F**), with model validity confirmed through 200 permutations. (**G**) Volcano plots display metabolic alterations between pre-NR vs post-NR (left) and pre-R vs post-R (right), with notable metabolites indicated by red (upregulated) and blue (downregulated) dots based on VIP score and Mann-Whitney *U* test thresholds. (**H**) A Venn diagram showing unique differential metabolites specific to the R group. (**I**) A bubble chart detailing the 20 enriched KEGG pathways associated with the different metabolites shown in panel **H**. (**J**) Heatmap illustrating significantly altered metabolites related to glycerophospholipid metabolism.

To assess the impact of LEEP surgery on the metabolomic profiles in the cervicovaginal microenvironment, we utilized targeted analysis of SCFAs alongside a non-targeted metabolomic approach to measure cervicovaginal secretions. The SCFA profiles demonstrated distinct clustering for the R group, as indicated by principal component analysis (PCA; ANOSIM, *P* = 0.386 for NR, *P* = 0.013 for R; Fig. 4A). Post-surgery, significant increases in acetic acid level were observed in the R group (two-sided unpaired *t*-test, *P* < 0.05), while the level in the NR group remained unchanged (Fig. 4B).

In total, we identified 1,387 metabolites through non-targeted metabolomic profiling. The metabolomic data indicated notable clustering specifically for the R group following surgery, as confirmed by PCA (ANOSIM, *P* = 0.001; Fig. 4C and E) and partial least squares discriminant analysis (PLS-DA; Fig. 4D and F). Further analysis revealed 28 metabolites in the NR group (21 upregulated and 7 downregulated) and 141 metabolites in the R group (75 upregulated and 66 downregulated), showing significant alterations (Fig. 4G). Importantly, 137 of these differential metabolites, either enriched or depleted, were exclusive to the R group (Fig. 1H), indicating their potential role in HPV clearance. Metabolite enrichment analysis highlighted a significant presence of differential metabolites involved in glycerophospholipid metabolism (Fig. 4I). Heatmap analysis indicated that most metabolites in this pathway were upregulated (Fig. 4J). Given the established roles of lipids in inflammation, our findings suggest their important involvement in post-surgical recovery.

### Pivotal correlations of disrupted cervicovaginal secretion bacteria and metabolites with HPV status

**Fig 5:**
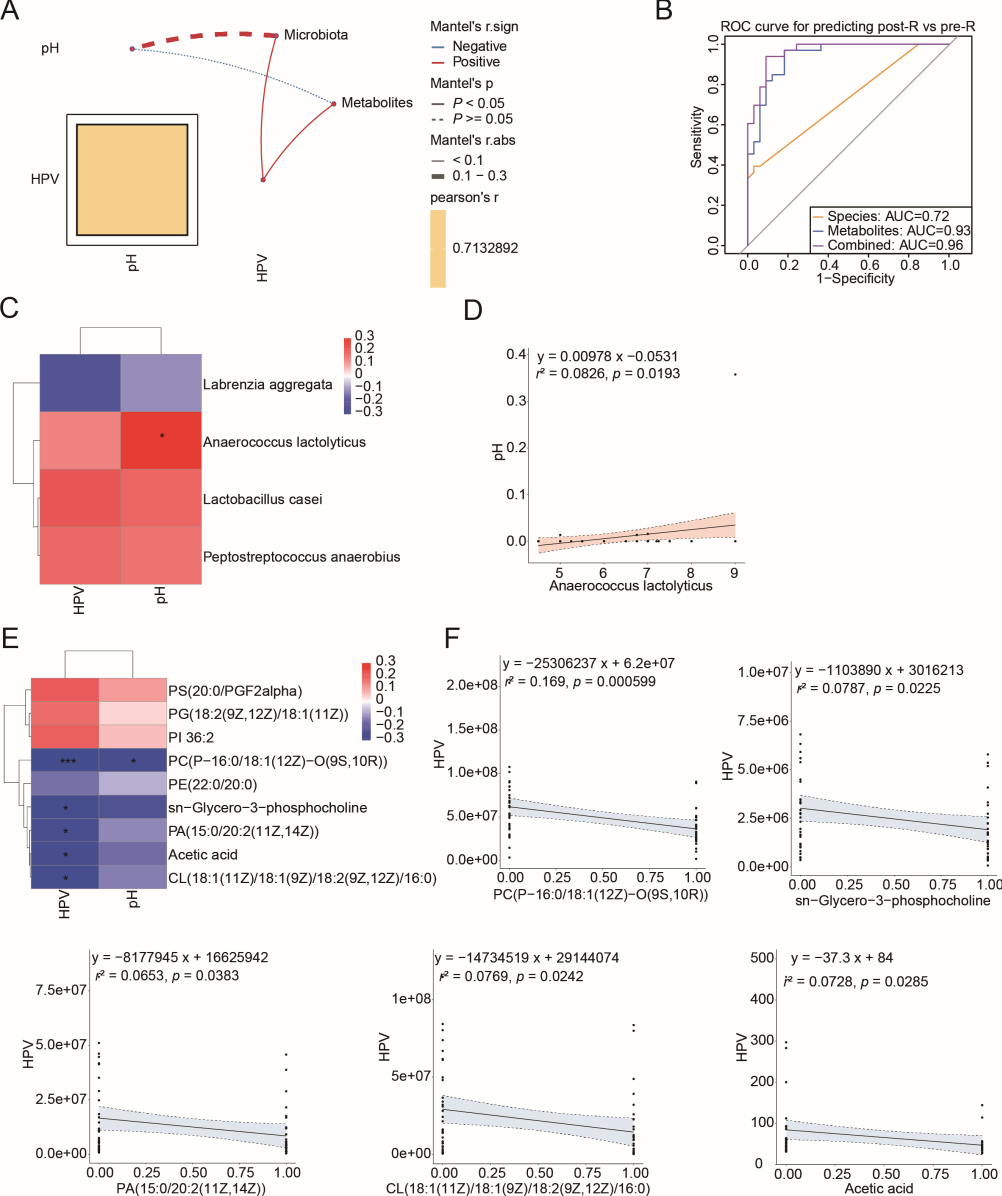
Correlation analysis among altered metabolites, cervicovaginal bacterial species, and clinical indices in the R group. (**A**) Correlation of clinical indices (HPV status and pH) with the nine altered metabolites related to glycerophospholipid metabolism and SCFAs, alongside four cervicovaginal DABs. (**B**) Receiver operating characteristic (ROC) curves displaying discriminatory signatures from the four bacterial species and nine metabolites for the cohort of 86 samples. (**C**) Pearson’s correlation analysis between DABs and clinical indices. ^*^P < 0.05, **P < 0.01, or ***P < 0.001. (**D**) Linear regression of *A. lactolyticus* against pH change. The solid black line indicates a statistically significant linear relationship (*P* < 0.05), and the shaded regions represent the 95% confidence intervals. Each black dot represents an individual sample. (**E**) Correlation analysis of altered metabolites with clinical indices. *P < 0.05, **P < 0.01, or ***P < 0.001. (**F**) Linear regression for the five metabolites and HPV status.

To explore the potential correlations between the four altered cervicovaginal secretion bacteria and nine modified metabolites associated with glycerophospholipid metabolism and SCFAs specific to the R group in relation to clinical indices, Pearson correlation analysis was performed. The results indicated significant correlations between entirely altered bacteria and metabolites with HPV clearance (*P* < 0.05), though no correlation was detected with changes in pH (*P* > 0.05), as substantiated by the Mantel test (Fig. 5A). We then assessed the efficacy of these bacterial and metabolomic markers in distinguishing patients before and after surgical treatment. Our predictive model revealed that bacterial profiles exhibited moderate sensitivity in identifying post-R patients, achieving an area under the curve (AUC) of 0.72 (Fig. 5B). The integration of bacterial species and metabolites improved classification accuracy, yielding an AUC of 0.96 compared to 0.72 when bacterial species were analyzed alone.

To further examine the individual associations between bacteria, metabolites, and clinical indices, Pearson correlation analysis was reiterated. Although no direct correlation between bacteria and HPV status was found, a significant positive relationship was noted between *Anaerococcus lactolyticus* and pH change (Fig. 5C and D). Additionally, five out of six metabolites—namely, PC(P-16:0/18:1(12Z)-O(9S,10R)), sn-glycero-3-phosphocholine, PA(15:0/20:2(11Z,14Z)), CL(18:1(11Z)/18:1(9Z)/18:2(9Z,12Z)/16:0), and acetic acid—elevated following therapy and were negatively associated with HPV status, as revealed by linear regression analysis (Fig. 5E and F). Notably, PC(P-16:0/18:1(12Z)-O(9S,10R)) also showed a negative correlation with pH change (Fig. 5E; Fig. S2A). These findings underscore an interaction between cervicovaginal bacteria, glycerophospholipid metabolism, and acetic acid in relation to HPV clearance and pH changes after LEEP surgery.

### Pivotal correlations of disrupted cervical tissue bacteria and metabolites with HPV status

**Fig 6:**
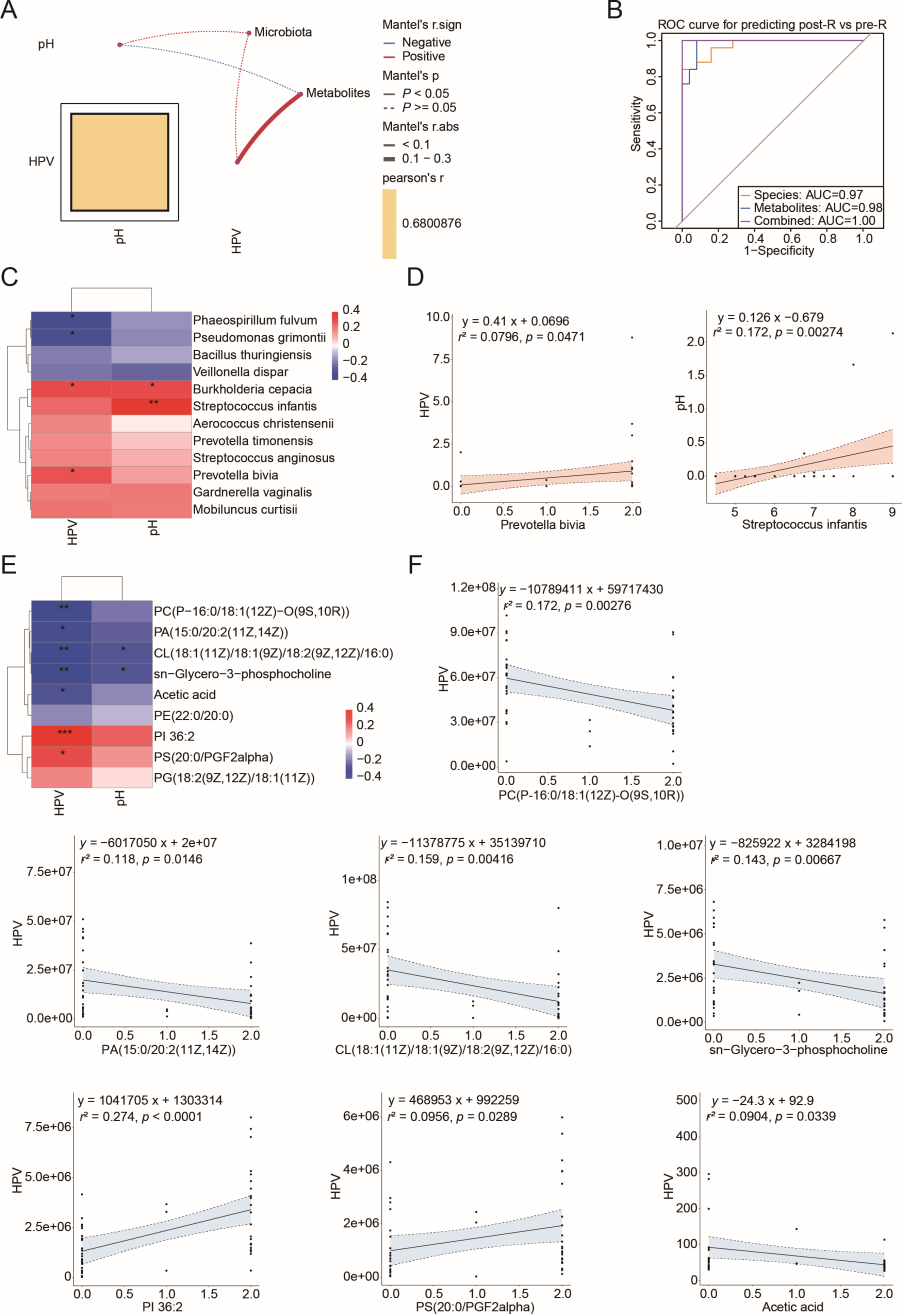
Correlation analysis among altered metabolites, cervical tissue bacterial species, and clinical indices in the R group. (**A**) Correlation of clinical indices (HPV status and pH) with the 9 altered metabolites related to glycerophospholipid metabolism and SCFAs, along with 12 cervical tissue DABs. (**B**) Receiver operating characteristic (ROC) curves constructed to display discriminatory signatures from the 12 bacterial species and 9 metabolites for the 68 sample cohort. (**C**) Pearson’s correlation analysis between DABs and clinical indices. *P < 0.05, **P < 0.01, or ***P < 0.001. (**D**) Linear regression analyses for *P. bivia* and HPV status, and *Streptococcus infantis* and pH change. The solid black line represents a statistically significant linear relationship (*P* < 0.05), while the shaded areas illustrate the 95% confidence intervals. Each black dot corresponds to an individual sample. (**E**) Correlation analysis of altered metabolites with clinical indices. *P < 0.05, **P < 0.01, or ***P < 0.001. (**F**) Linear regression for the seven indicated metabolites and HPV status.

To explore the correlations between the 12 altered cervical tissue bacteria and 9 altered metabolites associated with glycerophospholipid metabolism and SCFAs related to the R group, we employed Pearson’s correlation analysis. Our findings revealed significant associations between entirely altered metabolites and HPV status (*P* < 0.05), while no significant correlation was found with pH change (*P* > 0.05), as validated by the Mantel test (Fig. 6A). We subsequently assessed the potential of these bacterial and metabolomic markers in distinguishing patients before and after surgical intervention. Our predictive model showed that both bacterial and metabolic signatures had high sensitivity in identifying post-R patients, with AUC values of 0.97 and 0.98, respectively (Fig. 6B). Notably, the integration of bacterial species with metabolites significantly improved classification accuracy when compared to using bacterial species alone (AUC = 1.0 vs 0.97).

To further examine the relationships between individual bacteria, metabolites, and clinical indices, we conducted additional Pearson correlation analyses. We identified two positive associations: *P. bivia* with HPV and *Streptococcus infantis* with pH (Fig. 6C). Furthermore, seven out of nine metabolites—including PC(P-16:0/18:1(12Z)-O(9S,10R)), PA(15:0/20:2(11Z,14Z)), CL(18:1(11Z)/18:1(9Z)/18:2(9Z,12Z)/16:0), sn-glycero-3-phosphocholine, PI 36:2, PS(20:0/PGF2alpha), and acetic acid—showed significant associations with HPV status. Notably, CL(18:1(11Z)/18:1(9Z)/18:2(9Z,12Z)/16:0) and sn-glycero-3-phosphocholine were also related to changes in pH (Fig. 6E). Linear regression analysis further highlighted the interactions among microbiota, metabolites, HPV status, and pH change (Fig. 6D and F; Fig. S2B). These results suggest a significant interplay between cervical tissue bacteria, glycerophospholipid metabolism, and acetic acid in response to HPV clearance and pH alterations following LEEP surgery.

## DISCUSSION

Our analysis of 5R 16S rRNA sequences from cervicovaginal secretions and cervical tissue samples sheds light on the microbial diversity in these two distinct environments. At the phylum level, most identified phyla were common to both environments, reflecting a shared and diverse microbial ecosystem. However, only 7.3% of species overlapped, a divergence likely driven by two key factors. First, the externally exposed cervicovaginal region is more susceptible to microbial colonization, whereas the deeper cervical tissue, with its stronger immune and physiological barriers, harbors a more selective microbiota (29). Second, microenvironmental differences—such as pH, oxygen levels, and nutrient availability—between these niches likely promote the growth of distinct bacterial species, further explaining the observed compositional divergence (30). Interestingly, half of the 20 most abundant bacterial species were shared between the samples, suggesting common ecological interactions. Dominant species included various *Lactobacillus* species—such as *L. iners*, *L. crispatus*, *L. jensenii*, and *L. gasseri*—alongside potentially pathogenic anaerobes like *G. vaginalis*, *A. vaginae*, and certain species of *Prevotella* and *Streptococcus*. These findings align with existing literature (20, 28), reinforcing the notion that while there are notable differences in microbial composition, key species, especially *Lactobacillus* and specific symbiotic anaerobes, are essential to the ecology of both cervicovaginal secretions and cervical tissue.

The analysis of alpha diversity in cervicovaginal secretions and cervical tissues revealed significant variations in the NR group, while the R group exhibited stable alpha diversity. This suggests that changes in the local microbiota may hinder HPV clearance. In contrast, while beta diversity shifts were observed in cervical tissue, they remained stable in cervicovaginal secretions, indicating that surgical procedures have a direct effect on the tissue microenvironment. Notably, the NR group exhibited fewer significantly abundant microbial taxa compared to the R group, which demonstrated a greater number of significant changes, particularly in cervical tissues, underscoring the more pronounced impact of the procedure in this area. *L. iners* exhibited characteristics of both beneficial lactobacilli in healthy conditions and potential pathogens under certain dysregulated situations (16, 31). This species was strongly correlated with HPV clearance (32) and was more prevalent in the pre-NR group, suggesting its role in influencing HPV clearance. The R group experienced a reduction in normal bacterial populations, such as *L. casei*, *P. anaerobius*, and *A. lactolyticus* in cervicovaginal secretions post-surgery, indicating a disruption in cervicovaginal microbiota balance. Conversely, levels of harmful anaerobic or facultative anaerobic bacteria, including *G. vaginalis* and various species of *Prevotella* and *Streptococcus*, significantly decreased in cervical tissues after surgery, suggesting that the procedure promotes recovery of the cervical microbial community and aids in HPV clearance. These findings underscore that surgical interventions can substantially alter local microbiota, particularly in cervical tissues, which may influence HPV clearance, with *L. iners* playing a crucial role in this process.

*P. bivia* is a prevalent member of the nonpigmented *Prevotella* genus, which plays a significant role in obstetric and gynecologic infections (33). Research suggests that *P. bivia* in the vaginal microenvironment may contribute to persistent HPV infections, likely due to its production of high lipopolysaccharide concentrations and induction of various cytokines, which can disrupt vaginal microbial homeostasis (20, 34–36). Additionally, *Anaerococcus* species, including *A. lactolyticus*, have been isolated from vaginal discharges and ovarian abscesses (37). An increased abundance of *Anaerococcus* has been observed in the vaginal microbiota of cervical cancer patients (38) and the cervix and endometrium of those with endometrial cancer (39). Elevated *Anaerococcus* levels have also been linked to HPV infection and vaginal dysbiosis (38, 40, 41), potentially contributing to the development of CIN lesions (38). While certain *Anaerococcus* species, such as *Anaerococcus tetradius*, have been identified (11), specific studies on *A. lactolyticus* remain limited. Furthermore, *Streptococcus*, a β-hemolytic gram-positive bacterium, colonizes the lower genital tract of women (42) and has been recognized as an oral bacterial marker for cervical cancer (43). Its association with HPV infection and CIN has also been documented (44). These findings imply that *P. bivia* may contribute to dysbiosis in the vaginal microbiome related to HPV infection, potentially leading to increased abundance of *A. lactolyticus* and *S. infantis*. This rise in anaerobic bacteria may elevate vaginal pH, ultimately contributing to the progression of CIN.

Current research on the metabolic signatures of CIN following LEEP surgery is limited. Our metabolic profiling has revealed significant differences in the R group within both targeted and non-targeted metabolomics, indicating a shift in microbe-metabolite interactions in response to HPV clearance post-surgery. Notably, we observed dysregulation of SCFAs, particularly acetic acid, along with various glycerophospholipids following surgical intervention. SCFAs play a crucial role in anti-inflammatory responses through diverse signaling pathways (45). Specifically, our study linked acetic acid to HPV clearance and corroborated previous findings that it inhibits LPS-induced TNFα secretion from mononuclear cells and neutrophils (46, 47). Furthermore, lipid metabolism is integral to the inflammatory processes associated with various diseases, including CIN (48). Interestingly, SCFAs serve not only as substrates for lipid synthesis but also as regulatory factors in lipid metabolism (49, 50). For example, acetic acid is a key precursor in the synthesis of acetyl-CoA, palmitate, and stearate (51, 52). Since SCFAs are primarily produced by anaerobic bacteria (53), we hypothesize that LEEP surgery may enhance the activity of specific SCFA-producing bacteria, such as *P. bivia*. This enhancement could modulate glycerophospholipid levels and subsequently influence the inflammatory response, thereby supporting recovery from CIN and facilitating HPV clearance.

Our study has several limitations. First, the cohort size was restricted due to the small number of individuals available for follow-up after LEEP surgery. Future research should focus on larger, multicenter populations to validate these findings. Second, we were unable to obtain cervical tissue samples from certain patients, both pre- and post-surgery, resulting in a lower number of paired samples for 16S sequencing compared to cervicovaginal secretions. Finally, our conclusions are primarily based on omics data, underscoring the need for additional *in vitro* and *in vivo* experiments to achieve comprehensive validation.

In conclusion, our study sheds light on the intricate interplay between the cervicovaginal and cervical tissue microbiomes, along with the associated metabolomic profiles, in relation to HPV clearance following LEEP surgery in women diagnosed with CIN. Through a multi-omics approach, we identified distinct microbial communities and metabolic alterations associated with HPV status, highlighting specific bacterial species and metabolites that may influence the likelihood of HPV clearance. Notably, the presence of beneficial *Lactobacillus* species and the production of SCFAs, particularly acetic acid, were linked to successful HPV clearance, while certain anaerobic bacteria were more prevalent in patients with persistent infections. These findings underscore the potential role of the microbiome and its metabolic products in shaping patient outcomes post-LEEP and suggest avenues for future research aimed at developing therapeutic strategies, such as *Lactobacillus*-targeted probiotics or SCFA supplementation, to enhance HPV clearance.

## MATERIALS AND METHODS

### Sample collection and study design

A total of 80 participants, aged 20–50 and diagnosed with either cervical intraepithelial neoplasia I (*n* = 30, persisting for over 18 months) or CIN II/III (*n* = 50), were enrolled in this study between June 2023 and August 2024. However, as many patients from other regions underwent follow-up examinations locally at 6 months post-treatment, only 43 participants completed postoperative follow-up, including 11 with CIN I and 32 with CIN II/III. Participants were included based on the following criteria: (i) no pregnancy, lactation, or menstruation at the time of sample collection; (ii) no vaginal intercourse or vaginal cleansing within the previous 3 days; (iii) no antibiotic/probiotic use or barrier contraceptive methods within the last month; (iv) absence of HIV or hepatitis B/C infection; and (v) no prior history of endocrine or autoimmune disorders or any malignant tumors.

Eight swabs were used to collect cervicovaginal secretions from each participant. Four swabs were collected 6 months prior to the LEEP. The remaining four swabs were collected 6 months after the LEEP. During each visit, one swab was used for pH measurement and HPV genotyping, while the other three were allocated for analyzing the cervicovaginal microbiome, SCFAs, and the non-targeted metabolome. Additionally, cervical tissue samples were collected during either the LEEP procedure or colposcopy at each visit for cervical microbiome analysis. HPV DNA detection was conducted using the Hybribio HPV typing kit (Chaozhou Hybribio Biotechnology Corp, Chaozhou, China), as previously detailed (54). All samples were shipped on dry ice and stored at –80°C until processing.

### 5R 16S rRNA gene sequencing

DNA was extracted from frozen samples using the CTAB method in conjunction with the DP302-02 kit (TianGen, Beijing, China), in accordance with the manufacturer’s instructions. The sequencing of the 5R 16S rRNA was performed by LC-Bio Technology Co., Ltd (Hangzhou, China). For sample evaluation, a five-region amplification method was utilized as described in an earlier study (15). Sequencing took place on the Illumina NovaSeq6000 platform, where reads were demultiplexed by sample, filtered, and aligned to the five amplified regions based on the primer sequences. The short multiple regions framework approach was employed to consolidate read counts from these regions into a single profile by resolving a maximum likelihood estimation problem (55). The analysis referenced the GreenGenes database and applied the expectation maximization algorithm to assess microbial relative abundance. Data analysis integrity may be affected by issues such as low-quality sequences and primer splicing mistakes; hence, various filters were applied to detect and remove contamination, as previously described (15).

For microbial diversity analysis, we employed QIIME 1 (version 1.8.0) for alpha and beta diversity calculations, with visualizations conducted using the vegan package (version 2.6.2). Alpha diversity was measured using several indices, including Chao1, observed species, Shannon, and Simpson. Beta diversity was assessed through the Mann-Whitney *U* test based on Bray-Curtis distance. To analyze the relative abundance of bacterial species across different taxonomic levels, we used the ggplot2 package (version 3.4.0). The Mann-Whitney *U* test was also applied for differential analysis of microbial communities between groups, with a significance threshold set at *P* < 0.05. Additionally, receiver operating characteristic analysis was performed using the pROC package (version 1.18.5). Additional methodological details are provided in the Supplemental methods.

### Quantitative analysis of SCFAs

The metabolites were precipitated using an 80% methanol-water solution. Subsequently, a solution containing 1-ethyl-3-(3-dimethylaminopropyl) carbodiimide and 3-nitrophenylhydrazine was added to the sample to initiate the derivatization process. SCFA quantification was conducted by LC-Bio Technology Co., Ltd (Hangzhou, China). For liquid chromatography coupled with tandem mass spectrometry (LC-MS/MS) analysis, the target compounds were separated and quantified using an AB Sciex Jasper ultra-performance liquid chromatograph coupled with an AB SCIEX 4500MD triple quadrupole mass spectrometer. Separation was achieved on an Agilent Poroshell 120 EC-C_18_ column (3.0 × 150 mm, 2.7 µm). The mobile phases consisted of (A) pure water and (B) a methanol/acetonitrile mixture (vol/vol = 1:1), with an injection volume of 1 µL at a temperature of 40°C.

Mass spectrometric analysis was performed using an electrospray ionization Turbo Ion-Spray interface and operated in both positive and negative ion modes. The ion source parameters were optimized, with a turbo spray temperature of 400°C and an ion spray voltage of −3,000 V (in negative mode). Collision energies for the respective multiple reaction monitoring (MRM) transitions were fine-tuned, and a specific set of MRM transitions was closely monitored. For the raw data containing missing values, measurements below the limit of quantification were recorded as zero. Two-sided unpaired *t*-test was conducted to compare metabolite measurements between the two sample groups, designating differential metabolites as significant at *P* < 0.05. PCA was carried out using the “stats” package (version 4.3.2) in R to identify specific differences between the groups.

### Non-targeted metabolomic profiling of cervicovaginal secretions

Non-targeted metabolomic profiling of cervicovaginal secretions was conducted by LC-Bio Technology Co., Ltd (Hangzhou, China). Following metabolite extraction, samples were analyzed using LC-MS, and metabolomics data were preprocessed as previously described (56). PCA and PLS-DA were performed using metaX (version 1.4.16). Statistical significance for PCA and PLS-DA visualizations was assessed via the Mann-Whitney *U* test (based on Bray-Curtis distances) using the vegan R package. Differential metabolites were identified using a two-sided unpaired *t*-test, with significance thresholds set at variable importance in projection (VIP) > 1, *P* < 0.05, and fold change >1.2. Additional methodological details are provided in the Supplemental methods.

### Statistical analysis

Classified data are presented as counts or mean ± standard deviation (SD). For statistical analysis, the Chi-square test was employed for categorical variables, while the two-sided unpaired *t*-test was used for continuous variables. Inter-group comparisons were conducted using either the two-sided unpaired *t*-test or the Mann-Whitney *U* test, depending on the data distribution. Pearson correlations were calculated to generate a correlation heatmap among microbiota, metabolites, and clinical indicators, utilizing the tools provided by OmicStudio, available at https://www.omicstudio.cn/tool/62. The statistical analyses were carried out with GraphPad Prism 7.0 (GraphPad Software, CA, USA) and SPSS software (standard version 20.0; IBM). *P*-values are indicated as *P < 0.05, **P < 0.01, and ***P < 0.001.

## Data Availability

The 5R 16S rRNA sequencing data for the cervicovaginal secretions reported in this paper have been deposited in Genome Sequence Archive (GSA) with accession CRA022316. The 5R 16S rRNA sequencing data for the cervical tissue reported in this paper have been deposited in GSA with accession CRA022298. The non-targeted metabolomics data reported in this paper have been deposited in OMIX with accession OMIX008732. The targeted metabolomics data for SCFAs reported in this paper have been deposited in OMIX with accession OMIX008719. The completed STORMS (Strengthening the Organizing and Reporting of Microbiome Studies) checklist (57) for this study is provided at Zenodo with https://doi.org/10.5281/zenodo.15229660. The authors declare that all the data supporting the findings of this study are available within the paper or from the corresponding authors upon request.

## SUPPLEMENTAL MATERIAL

Supplemental figures msystems.00511-25-s0001.docx Figures S1 and S2.

Supplemental text msystems.00511-25-s0002.docx Supplemental methods.

Supplemental tables msystems.00511-25-s0003.xlsx Tables S1 and S2.

ASM does not own the copyrights to Supplemental Material that may be linked to, or accessed through, an article. The authors have granted ASM a non-exclusive, world-wide license to publish the Supplemental Material files. Please contact the corresponding author directly for reuse.
