# Supplementary material for: Microbiome and metabolomic changes associated with HPV clearance in women undergoing local excisional treatment for cervical intraepithelial neoplasia
Source: mSystems. 2025 May 20;10(6):e00511-25. doi: 10.1128/msystems.00511-25 (PMC12172484; doi:10.1128/msystems.00511-25)
Supplement: Supplemental figures — Figures S1 and S2. [file msystems.00511-25-s0001.docx]

**Microbiome and metabolomic changes associated with HPV clearance in women undergoing local excisional treatment for cervical intraepithelial neoplasia**

Xiaowen Pu^1^, Jingjing Wang^1^, Zhengrong Gu^1^, Hongfeng Ao^2,*^, Chao Li^3,*^

^1^Department of Gynecology, Shanghai First Maternity and Infant Hospital, School of Medicine, Tongji University, Shanghai 200092, China

^2^Department of Pathology, Shanghai Fengxian District Central Hospital, Shanghai Jiao Tong University Affiliated Sixth People’s Hospital South Campus, Shanghai 201499, China

^3^Shanghai Key Laboratory of Maternal Fetal Medicine, Shanghai Institute of Maternal-Fetal Medicine and Gynecologic Oncology, Clinical and Translational Research Center, Shanghai First Maternity and Infant Hospital, School of Medicine, Tongji University, Shanghai 200092, China

^*^**Correspondence to:**

Hongfeng Ao, aohongfeng@126.com, Department of Pathology, Shanghai Fengxian District Central Hospital, Shanghai Jiao Tong University Affiliated Sixth People’s Hospital South Campus, No. 6600, Nanfeng Highway, Shanghai 201499, China

Chao Li, lichao126688@126.com, Shanghai Key Laboratory of Maternal Fetal Medicine, Shanghai Institute of Maternal-Fetal Medicine and Gynecologic Oncology, Clinical and Translational Research Center, Shanghai First Maternity and Infant Hospital, School of Medicine, Tongji University, No. 2699 West Gaoke Road, Shanghai 200092, China

**
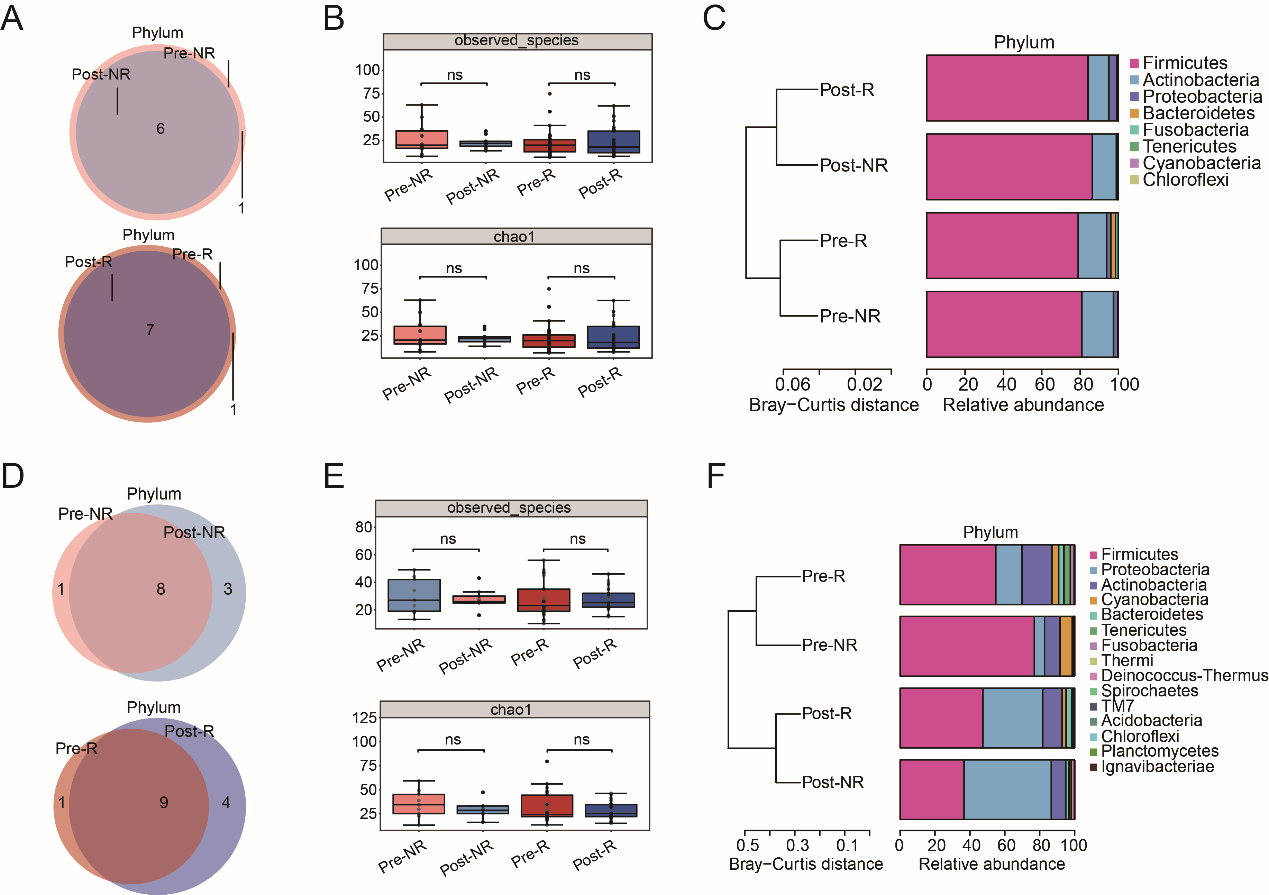
**

**Figure S1. Overview the results for the 5R 16S rRNA gene sequencing from cervicovaginal secretion and cervical tissue samples.**

(A and D) Venn diagrams showing bacterial phyla in cervicovaginal secretion (A) and cervical tissue (D) before and after LEEP, contrasting post-NR vs pre-NR (top) and post-R vs pre-R (bottom). (B and E) Alpha diversity assessment using Observed features and Chao1 indices for cervicovaginal secretion (B) and cervical tissue (E), with *P*-values calculated via the Mann-Whitney *U* test. (C and F) Hierarchical clustering on average relative proportions of phyla across the four groups for cervicovaginal secretion (C) and cervical tissue (F), based on Bray-Curtis distances.


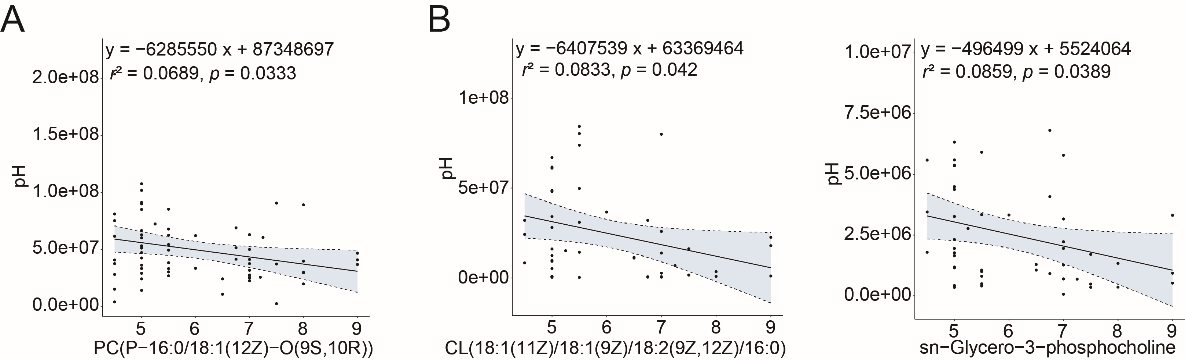


**Figure S2.** Linear regression illustrating the relationship between PC(P-16:0/18:1(12Z)-O(9S,10R)) and pH change (refer to Figure 5F) (A), or CL(18:1(11Z)/18:1(9Z)/18:2(9Z,12Z)/16:0), sn-Glycero-3-phosphocholine and pH change (refer to Figure 6F) (B). Solid black lines indicate significant linear relationships (*P* < 0.05), with shaded areas representing 95% confidence intervals. Each black dot corresponds to an individual sample.
